# Spicy food consumption and risk of gastrointestinal-tract cancers: findings from the China Kadoorie Biobank

**DOI:** 10.1093/ije/dyaa275

**Published:** 2021-01-23

**Authors:** Wing Ching Chan, Iona Y Millwood, Christiana Kartsonaki, Huaidong Du, Yu Guo, Yiping Chen, Zheng Bian, Robin G Walters, Jun Lv, Pan He, Chen Hu, Liming Li, Ling Yang, Zhengming Chen, for the China Kadoorie Biobank (CKB) Collaborative Group

**Affiliations:** 1 Clinical Trial Service Unit and Epidemiological Studies Unit (CTSU), Nuffield Department of Population Health, University of Oxford, Oxford, UK; 2 Medical Research Council Population Health Research Unit, Nuffield Department of Population Health, University of Oxford, Oxford, UK; 3 Chinese Academy of Medical Sciences, Beijing, China; 4 NCDs Prevention and Control Department, Huixian Center for Disease Control and Prevention, Henan, China; 5 Department of Epidemiology and Biostatistics, School of Public Health, Peking University, Beijing, China

**Keywords:** Spicy food, chilli peppers, gastrointestinal cancers, digestive cancers, prospective cohort studies

## Abstract

**Background:**

Previous case–control studies have reported positive associations of spicy food consumption with risks of certain gastrointestinal-tract (GI) cancers. However, there is no prospective evidence on such associations, particularly from China, where there are high incidence rates of GI cancers and spicy food is widely consumed.

**Methods:**

The prospective China Kadoorie Biobank study recruited >512 000 adults aged 30–79 years from 10 areas in China during 2004–2008; 2350 oesophageal, 3350 stomach and 3061 colorectal incident cancer cases were recorded by 1 January 2017, after a median of 10.1 years of follow-up. Cox regression yielded adjusted hazard ratios (HRs) for each cancer associated with spicy food intake.

**Results:**

Overall, 30% of participants reported daily spicy food consumption at baseline. Spicy food consumption was inversely associated with oesophageal cancer risk, with adjusted HRs of 1.00, 0.88, 0.76, 0.84 and 0.81 for those who never/rarely consumed (reference) and consumed monthly, 1–2 days/week, 3–5 days/week and 6–7 days/week, respectively (*p*_trend_ < 0.002). The association remained similar after excluding the first 3 years of follow-up but appeared stronger in participants who did not smoke or drink alcohol regularly (*p*_trend_ < 0.0001). The corresponding HRs for stomach cancer were 1.00, 0.97, 0.95, 0.92 and 0.89 (*p_trend_* = 0.04), with the association disappearing after excluding the first 3 years of follow-up. For colorectal cancer, the HRs were 1.00, 1.00, 0.95, 0.87 and 0.90, respectively (*p*_trend_ = 0.04) and the inverse association appeared to be restricted to rectal rather than colon cancer (*p*_heterogeneity_ = 0.004). The types and strength of spice used showed little additional effects on these associations.

**Conclusion:**

In Chinese adults, higher spicy food consumption was associated with lower risks of certain GI cancers, particularly among individuals who never smoked or drank alcohol regularly.

## Introduction

Worldwide, gastrointestinal-tract (GI) cancers accounted for over one-fifth of all cancer incidence and death in 2018.^1^ In China, the rates of oesophageal and stomach cancers are particularly high and account for over half of the global burden of these two cancers.^1–3^ Notably, ∼90% of oesophageal cancers in China are squamous cell carcinoma (ESCC), in contrast to Western countries, where adenocarcinoma (EAC) is the predominant subtype.^4^ Coupled with a rising rate of colorectal cancer, GI cancers were responsible for >1.3 million new cancer cases and 0.9 million deaths in China in 2018.^5^

Diet plays an important role in the aetiology of many GI cancers. Spicy food (i.e. dishes or food made with chilli peppers or sauce^6^) is widely consumed in many parts of the world, including certain regions of China.^7^ Chilli peppers are rich in the bioactive component capsaicin, which has been shown, though not consistently, by *in vitro* and *in vivo* experiments to have various anti-cancer properties such as inhibiting the proliferation of, and inducing apoptosis in, human stomach and colorectal cancer cells.^8^^,^^9^ However, capsaicin has also been reported to induce stomach tumours in mice^10^ and to increase the migratory capability of colorectal cancer cells.^11^ Higher chilli intake has also been shown in cross-sectional epidemiological studies to be associated with a lower prevalence of obesity and lower serum cholesterol and inflammatory-marker levels, which are potential risk factors for GI cancers.^12^^,^^13^

**Key Messages**

- To our knowledge, all existing evidence on spicy food intake and cancers of the gastrointestinal-tract came from small studies with a case–control study design, with important methodological limitations such as recall bias, reverse causation and limited statistical power.
- In a large Chinese cohort, we prospectively examined the relationships between spicy food consumption and the risks of developing oesophageal, stomach and colorectal cancers.
- Our study found that the frequency of spicy food intake was inversely associated with oesophageal cancer risk; this association appeared robust, persisting after attempts to minimize reverse causation and residual confounding by smoking and/or alcohol-drinking.
- Relationships between spicy food intake and stomach and colorectal cancer risk were less clear, with some evidence of weak inverse associations.

Previous epidemiological studies on spicy food consumption and risks of GI cancers, conducted predominantly in Asian populations, have reported mostly null or positive associations.^6^ However, these previous studies have been constrained by the use of the case–control design, small numbers of events (typically <300 cases),^14–18^ crude exposure assessment (e.g. ‘seldom’ vs ‘often’)^19–21^ and/or inadequate adjustment for important confounders, especially smoking and/or alcohol-drinking.^15^^,^^16^^,^^19^

To address the evidence gap, we present detailed analyses, building on our previous report of spicy food and mortality from total cancer and other diseases,^22^ on the associations of spicy food consumption with incidence of three major GI (i.e. oesophageal, stomach and colorectal) cancers in the prospective China Kadoorie Biobank (CKB) study.

## Methods

### Study population

CKB is a prospective cohort study of 512 715 participants, aged 30–79 years at enrolment, from 5 urban and 5 rural areas in China. Details of the CKB study design have been described elsewhere.^23^^,^^24^ In brief, participant recruitment took place in 2004–2008 and the baseline assessment consisted of an interviewer-administered electronic questionnaire (on socio-demographics, medical history, diet and lifestyle), physical measurements (e.g. anthropometrics) and blood-sample collection. Two resurveys were conducted in late 2008 and 2013–2014, respectively, amongst ∼5% of randomly selected, surviving participants. Ethics approval from the Oxford University Tropical Research Ethics Committee, the Chinese Centre for Disease Control and Prevention (CDC) Ethical Review Committee and the local CDC of each study area were obtained, and all participants provided written informed consent.

### Assessment of spicy food consumption

In CKB, spicy food intake refers to the direct consumption of fresh chilli peppers; the addition of fresh/dried chilli peppers, chilli oil/sauce/paste, curry or other ‘hot’ spices when cooking; or the addition of chilli oil/sauce/paste to food when eating. At baseline and resurveys, participants were asked about their frequency of spicy food consumption in the past month (never/almost never, only occasionally, 1–2 days/week, 3–5 days/week or 6–7 days/week). Amongst those who consumed spicy food at least once per week (defined as ‘regular consumers’), additional information on the age at which they started eating spicy food regularly, the strength of the spice preferred (weak, moderate, strong) and the main sources of spice typically used (fresh chilli pepper, dried chilli pepper, chilli sauce, chilli oil or others/don’t know) was collected. The duration of regular spicy food intake was derived by calculating the difference between the participants’ baseline age and the age at which they started eating spicy food regularly. To evaluate the reproducibility of spicy food consumption responses at baseline, ∼1000 participants were reassessed within 12 months (mean 5.4 months) after baseline.

### Follow-up and outcome measures

Participants were followed up via record linkage, using their unique national identification number, with local death and disease registries and nationwide health-insurance databases (which cover >96% of CKB participants), supplemented by annual active follow-up to minimize loss to follow-up.^23^ All fatal and non-fatal events were coded using the International Classification of Disease 10th Edition (ICD-10) by trained staff. Event adjudication was completed in a subset of participants, by reviewing medical notes to verify diagnoses and to obtain further clinical information (e.g. cancer sub-site and pathology subtype).

The main outcomes examined in the present study were incident oesophageal cancer (ICD-10: C15), stomach cancer (C16), colorectal cancer (C18–C20) and the three cancers combined (‘total GI cancers’). We also separately explored associations with cancer subtypes and sub-sites, using the preliminary data from event adjudication. Participants contributed person-years from their enrolment date until their date of the outcome of interest, death (from any cause), loss to follow-up or the study end date (31 December 2016 for this present study), whichever came first.

### Statistical analysis

Participants with a self-reported history of cancer (*n* = 2578) or negative values of spicy food consumption duration (*n* = 36) at baseline were excluded, leaving 510 101 for the present study. Age-, sex- and region-standardized prevalence or mean values of baseline characteristics were calculated across the five frequency levels of spicy food consumption. Linear regression was used to assess the associations between spicy food intake and measures of adiposity, serum lipids and inflammatory markers, with adjustment for socio-demographic, lifestyle and dietary factors. Cox regression was used to estimate the hazard ratios (HRs) and 95% confidence intervals (CIs) for each cancer in association with baseline measures of spicy food consumption. Models were stratified by age-at-risk (10-year bands) and sex, and adjusted for study areas, family history of cancer, education, household income, smoking, alcohol consumption, physical activity [in metabolic equivalent of task (MET) hours per day] and intake of fruits, meat, dairy products and preserved vegetables. Stratification by age-at-risk bands allows the baseline hazard to be different for each band whilst estimating a single HR. Among regular consumers of spicy food, the effects of duration, starting age, spice strength and spice type were examined, with and without further adjustment for consumption frequency. Analyses of the duration of spicy food consumption were additionally adjusted for baseline age. Based on the adjusted HRs and the formula described by Liu *et al.*,^25^ cancer incidence rates (per 100 000 person-years) were calculated across categories of spicy food consumption frequency.^25^

Subgroup analyses by smoking and/or alcohol-drinking status were performed to examine potential effect modifications, with further subgroup analyses conducted by sex (only ∼3% of CKB women were ever-regular smokers/drinkers, hence this group was omitted). For oesophageal cancer, subgroup analyses were also conducted by high- (Huixian, a high-risk area where 52% of oesophageal cancer cases in CKB occurred) and low-incidence areas (the other nine combined). Adiposity measures may act as mediators^12^^,^^26^ and were adjusted for in a sensitivity analysis. Other sensitivity analyses included stratifying by regions (which may better account for confounding by regions); the exclusion of regions with extremely high intake levels (i.e. Hunan and Sichuan); and, to minimize the effect of reverse causation, the exclusion of: (i) participants with a history of peptic ulcers; and (ii) participants with any prior chronic diseases and the first 3 years of follow-up data.

When comparing HRs from multiple categories of exposure, the floating-absolute-risk method was used to calculate 95% CIs, allowing direct comparisons across categories and not only with the reference group.^27^^,^^28^ Trends were tested by fitting the ordinal spicy food variables as continuous in the models. The proportional-hazards assumption for the Cox model was assessed by comparing the HRs for the first and second half of the follow-up period. All analyses were performed using SAS (version 9.4) and R (version 3.3.3).

## Results

Of the 510 101 participants included, the mean (SD) baseline age was 52.0 (10.7) years, 59% were female and 44% resided in urban areas. Overall, 30.1% of participants reported consuming spicy food 6–7 days/week (defined as ‘daily-consumers’) in the past month, with a similar proportion (32.7%) reporting no or rare consumption (defined as ‘non-consumers’) (Table 1). Spicy food consumption varied notably across the 10 study areas; two-thirds of all daily-consumers were from two rural areas (i.e. Hunan and Sichuan), where 99.3% and 68.5% of participants were daily-consumers, respectively (Supplementary Figure 1, available as Supplementary data at *IJE* online). Weighted Kappa between responses at baseline and reassessment within 12 months ranged from 0.4 to 0.6 for the various spicy food measures, indicating moderately good reproducibility (Supplementary Table 1, available as Supplementary data at *IJE* online).

**Table 1:** Baseline characteristics of study participants by frequency of spicy food consumption^a^

	Frequency of spicy food consumption					
	Never/rarely	Monthly	1–2 days/wk	3–5 days/wk	6–7 days/wk	All participants
	(*N* = 166 972)	(*N* = 126 923)	(*N* = 32 934)	(*N* = 29 677)	(*N* = 153 595)	(*N* = 510 101)
**Socio-demographic factors**						
Age, year	55.3 ± 10.8	52.3 ± 10.5	50.1 ± 10.2	49.8 ± 10.0	49.5 ± 10.3	52.0 ± 10.7
Female, %	59.7	55.6	53.6	54.3	53.8	59.0
Urban, %	52.3	56.3	58.4	58.6	18.3	44.1
Educational level, %						
No formal education	20.3	18.3	16.7	17.5	17.0	18.6
Primary school	33.1	31.7	31.6	30.1	31.4	32.2
Middle school	26.0	28.3	28.2	29.4	29.7	28.3
High school or above	20.5	21.7	23.4	23.0	21.9	20.9
Household income (Yuan/year), %						
<10 000	34.3	28.7	27.7	26.8	25.9	28.2
10 000–19 999	28.1	28.1	28.4	29.5	29.3	29.0
20 000–34 999	21.6	24.7	25.1	23.7	24.5	24.7
≥35 000	15.9	18.6	18.8	20.0	20.3	18.0
**Lifestyle factors** ^b^						
Ever-regular smokers in men,%	69.0	71.7	76.8	77.0	81.4	74.3
Ever-regular smokers in women,%	2.7	2.8	3.3	4.1	4.8	3.3
Ever-regular alcohol-drinkers in men,%	36.2	40.5	45.3	50.3	53.9	41.9
Ever-regular alcohol-drinkers in women,%	1.9	2.7	3.2	3.7	5.1	2.9
Ever-regular tea consumption,%	31.7	32.3	36.5	38.5	41.2	35.6
Total physical activity, MET-hr/day	20.4 ± 14.6	21.2 ± 14.2	21.1 ± 13.4	21.4 ± 13.5	21.3 ± 12.9	21.1 ± 13.9
**Physical measurements**						
Body mass index, kg/m²	23.4 ± 3.4	23.7 ± 3.4	23.8 ± 3.3	23.9 ± 3.4	24.0 ± 3.3	23.7 ± 3.4
Waist circumference, cm	79.4 ± 9.9	80.3 ± 9.8	80.6 ± 9.8	80.7 ± 9.8	81.0 ± 9.4	80.3 ± 9.8
Systolic blood pressure, mmHg	130.7 ± 21.9	131.4 ± 21.1	130.4 ± 20.6	130.6 ± 20.8	131.3 ± 20.8	131.1 ± 21.3
**Regular dietary intake, %** ^c^						
Fresh fruits	28.3	28.9	31.5	30.7	32.8	28.2
Fresh vegetables	99.0	98.3	95.7	97.9	98.9	98.3
Preserved vegetables	20.7	20.8	24.7	24.2	28.4	22.6
Meat	44.3	46.9	49.8	48.3	51.2	47.2
Fish	7.7	8.8	9.7	10.5	10.9	8.9
Dairy	12.8	12.2	12.8	12.7	12.7	11.8
Eggs	23.0	22.5	23.5	24.4	28.0	24.4
**Self-reported medical history, %**						
Had peptic ulcer	5.3	4.3	4.0	3.9	3.3	3.9
Had any prior chronic diseases^d^	25.7	24.6	23.3	23.2	21.9	22.9
Family history of cancer	17.2	17.1	17.6	18.8	17.8	17.0

wk, week; MET-hr/day, metabolic equivalents of task per hours per day.

a Plus–minus values are mean±SD. Data were directly standardized to the age, sex and area structure of the study population where appropriate (unless otherwise specified).

b Ever-regular smokers include current- and ex-regular smokers; ever-regular alcohol/tea drinkers include current-regular, ex-regular and reduced-intake drinkers.

c Regular dietary intake refers to consumption on ≥4 days/week.

d Chronic diseases include chronic heart diseases, stroke/transient ischaemic attack, hypertension, diabetes, peptic ulcers, cirrhosis and kidney diseases (participants with prior cancer were not included in this study).

Individuals with more frequent spicy food consumption were more likely to be male, younger, have higher levels of income and be current-regular smokers or alcohol-drinkers and less likely to have a history of peptic ulcer or other chronic diseases (Table 1). More frequent consumers also had higher levels of adiposity measurements but mean levels of blood lipids and inflammatory markers (e.g. LDL-C, HDL-C, fibrinogen) were broadly similar across spicy food consumption frequencies (Supplementary Table 2, available as Supplementary data at *IJE* online). Amongst regular consumers, those with more frequent consumption typically started at a younger age, consumed spice for a longer duration (after adjusting for baseline age), preferred stronger spice intensity and consumed multiple types of spicy food (Supplementary Table 3, available as Supplementary data at *IJE* online).

During 5.1 million person-years of follow-up (median duration = 10.1 years), >20 600 incident cancer cases were recorded, of which 2350, 3350 and 3061 were oesophageal, stomach and colorectal cancers, with corresponding incidence rates of 46.4, 66.2 and 60.5 per 100 000 person-years. After adjusting for socio-demographic, lifestyle and other dietary factors, the frequency of spicy food consumption was inversely associated with the risks of GI cancers (Table 2). For oesophageal cancer, compared with non-consumers, the adjusted HRs were 0.88, 0.76, 0.84 and 0.81 for those who consumed spicy food monthly, 1–2 days/week, 3–5 days/week and 6–7 days/week, respectively (*p*_trend_ < 0.002). A similar association was observed when analyses were restricted to confirmed squamous-cell-carcinoma cases, despite having a non-significant trend possibly due to small case numbers (Supplementary Table 4, available as Supplementary data at *IJE* online). The corresponding HRs for stomach cancer were 1.00, 0.97, 0.95, 0.92 and 0.89 (*p*_trend_ = 0.04), with a significant inverse association observed for cardia (*p*_trend_ = 0.04) but not for non-cardia (*p*_trend_ = 0.2) cancer, although a test for heterogeneity showed no statistically significant difference (*p*_heterogeneity_ = 0.2). For colorectal cancer, the HRs were 1.00, 1.00, 0.95, 0.87 and 0.90, respectively (*p*_trend_ = 0.04) and the inverse association appeared to be restricted to rectum rather than colon cancer (*p*_heterogeneity_ = 0.004) (Table 2 and Supplementary Table 4, available as Supplementary data at *IJE* online). The frequency of spicy food intake was also inversely associated with oesophageal cancer mortality, but not with stomach or colorectal cancer mortality (Supplementary Table 5, available as Supplementary data at *IJE* online).

**Table 2:** Incidence rates and adjusted hazard ratios for gastrointestinal cancers according to spicy food consumption frequency

	Frequency of spicy food consumption					
	Never/rarely	Monthly	1–2 days/wk	3–5 days/wk	6–7 days/wk	
	(*N* = 166 972)	(*N* = 126 923)	(*N* = 32 934)	(*N* = 29 677)	(*N* = 153 595)	*p*_trend_
**Oesophageal cancer**						
No. of events	1078	621	83	82	486	
Age, sex, region-adjusted incidence rate	49.51	43.60	38.51	43.90	44.94	
Hazard ratio (95% CI) (Model 1)	1.00 (0.93–1.07)	0.92 (0.85–0.99)	0.83 (0.66–1.03)	0.93 (0.75–1.16)	0.95 (0.83–1.08)	0.3
Hazard ratio (95% CI) (Model 2)	1.00 (0.93–1.07)	0.88 (0.81–0.94)	0.76 (0.61–0.94)	0.84 (0.67–1.05)	0.82 (0.72–0.94)	0.003
Hazard ratio (95% CI) (Model 3)	1.00 (0.93–1.07)	0.88 (0.82–0.95)	0.76 (0.61–0.94)	0.84 (0.67–1.04)	0.81 (0.71–0.93)	0.002
**Stomach cancer**						
No. of events	1432	901	212	183	622	
Age, sex, region-adjusted incidence rate	69.33	66.03	64.34	63.16	60.77	
Hazard ratio (95% CI) (Model 1)	1.00 (0.94–1.06)	0.97 (0.91–1.04)	0.96 (0.84–1.10)	0.94 (0.81–1.08)	0.90 (0.81–1.00)	0.08
Hazard ratio (95% CI) (Model 2)	1.00 (0.94–1.07)	0.97 (0.91–1.03)	0.94 (0.82–1.08)	0.92 (0.79–1.06)	0.88 (0.79–0.98)	0.03
Hazard ratio (95% CI) (Model 3)	1.00 (0.94–1.07)	0.97 (0.91–1.03)	0.95 (0.83–1.08)	0.92 (0.79–1.06)	0.89 (0.80–0.99)	0.04
**Colorectal cancer**						
No. of events	1194	810	180	144	733	
Age, sex, region-adjusted incidence rate	61.75	62.58	59.39	54.78	57.63	
Hazard ratio (95% CI) (Model 1)	1.00 (0.94–1.07)	1.01 (0.94–1.08)	0.95 (0.82–1.10)	0.88 (0.74–1.03)	0.92 (0.83–1.03)	0.09
Hazard ratio (95% CI) (Model 2)	1.00 (0.93–1.07)	1.01 (0.94–1.07)	0.95 (0.82–1.10)	0.87 (0.74–1.02)	0.90 (0.81–1.01)	0.05
Hazard ratio (95% CI) (Model 3)	1.00 (0.93–1.07)	1.00 (0.94–1.07)	0.95 (0.82–1.10)	0.87 (0.73–1.02)	0.90 (0.80–1.01)	0.04
**Total (the above cancers combined)** ^a^						
No. of events	3528	2200	461	385	1773	
Age, sex, region-adjusted incidence rate	174.25	163.08	159.06	151.95	154.99	
Hazard ratio (95% CI) (Model 1)	1.00 (0.96–1.04)	0.95 (0.91–0.99)	0.93 (0.85–1.02)	0.89 (0.80–0.98)	0.91 (0.85–0.97)	0.003
Hazard ratio (95% CI) (Model 2)	1.00 (0.96–1.04)	0.94 (0.90–0.98)	0.91 (0.83–0.99)	0.86 (0.77–0.95)	0.86 (0.81–0.92)	<0.0001
Hazard ratio (95% CI) (Model 3)	1.00 (0.96–1.04)	0.94 (0.90–0.98)	0.91 (0.83–0.99)	0.85 (0.77–0.94)	0.86 (0.80–0.92)	<0.0001

wk, week. Rates are expressed in no./100 000 person-years.

Model 1: stratified by age-at-risk (10-year bands) and sex, and adjusted for study area, education level, household-income level and family history of cancer.

Model 2: additionally adjusted for smoking status, alcohol consumption and physical activity (MET-hr/day).

Model 3: additionally adjusted for dietary factors (consumption of fruits, meat, dairy, preserved vegetables) (plus tea consumption and temperature for oesophageal cancer and total gastrointestinal-tract cancers).

a This endpoint is the first incident gastrointestinal-tract cancer (which could be either oesophageal, stomach or colorectal).

**Figure 1:**
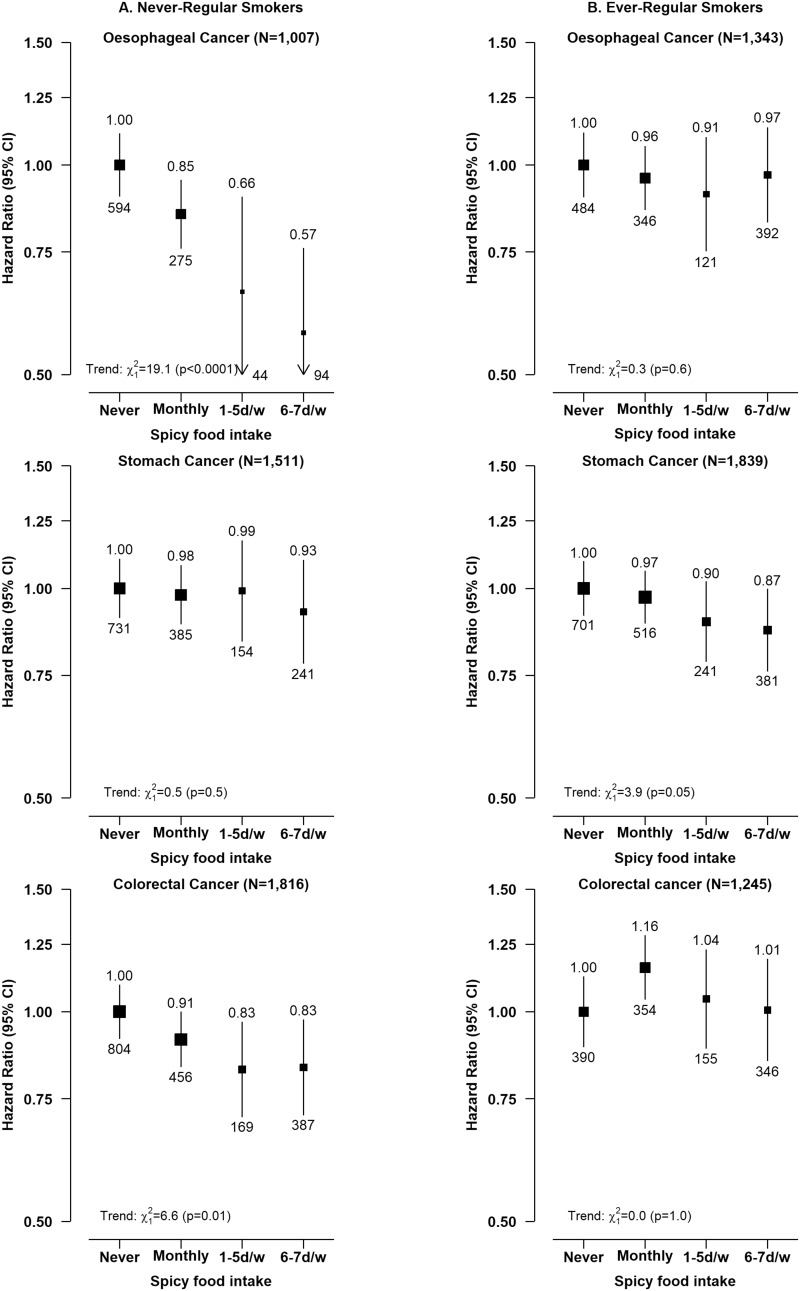
Adjusted hazard ratios (HRs) for gastrointestinal-tract cancers by frequency of spicy food intake in never-regular and ever-regular smokers. Analyses were stratified by age-at-risk (10-year bands) and sex, and adjusted for region, education level, household-income level, family history of cancer, alcohol consumption, physical activity and consumption of fruits, meat, dairy and preserved vegetables (plus tea consumption and temperature for oesophageal cancer). Trends were obtained by fitting ordinal variables in the Cox models as continuous. The size of each square is inversely proportional to the variance of its log-HR. The HR and number of events for each category are presented above and below the vertical line, respectively.

**Figure 2:**
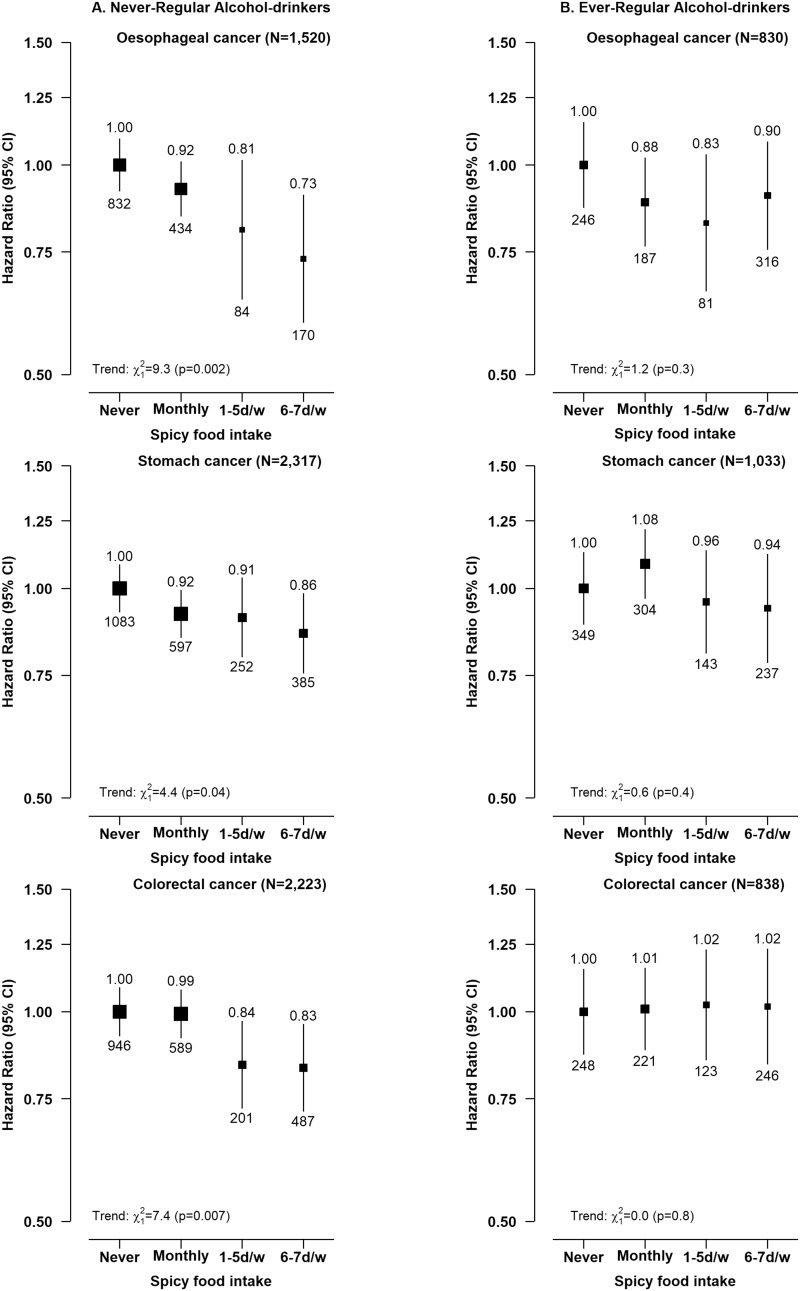
Adjusted hazard ratios (HRs) for gastrointestinal-tract cancers by frequency of spicy food consumption in never-regular and ever-regular alcohol-drinkers. Analyses were stratified by age-at-risk (10-year bands) and sex, and adjusted for region, education level, household-income level, family history of cancer, smoking status, physical activity and consumption of fruits, meat, dairy and preserved vegetables (plus tea consumption and temperature for oesophageal cancer). Trends were obtained by fitting ordinal variables in the Cox models as continuous. The size of each square is inversely proportional to the variance of its log-HR. The HR and number of events for each category are presented above and below the vertical line, respectively.

**Figure 3:**
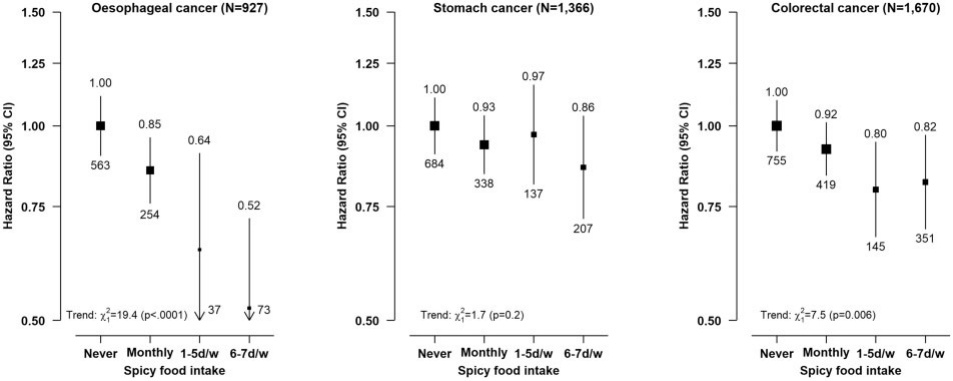
Adjusted hazard ratios (HRs) for gastrointestinal-tract cancers by frequency of spicy food consumption in participants who never smoked or drank regularly. Analyses were stratified by age-at-risk (10-year bands) and sex, and adjusted for regions, education level, household-income level, family history of cancer, physical activity and consumption of fruits, meat, dairy and preserved vegetables (plus tea consumption and temperature for oesophageal cancer). Trends were obtained by fitting ordinal variables in the Cox models as continuous. The size of each square is inversely proportional to the variance of its log-HR. The HR and number of events for each category are presented above and below the vertical line, respectively.

When stratified by smoking status, frequency of spicy food intake remained significantly and inversely associated with oesophageal cancer risk in never-regular, but not in ever-regular, smokers (*p*_interaction_ < 0.001; Figure 1). Compared with non-consumers, the adjusted HRs for daily-consumers were 0.57 (95% CI 0.43–0.77) and 0.97 (0.80–1.17) amongst never- and ever-regular smokers, respectively (Figure 1). For stomach and colorectal cancers, there was no similar effect modification by smoking status (*p*_interaction_ = 0.93; Figure 1). When stratified by alcohol-drinking status, the risk estimates appeared greater among never-regular than among ever-regular alcohol-drinkers for all three cancers, although the formal test for interaction was only statistically significant for oesophageal cancer [HR for daily vs non-consumption = 0.73 (0.59–0.92) in never-regular and 0.90 (0.72–1.13) in ever-regular drinkers; *p*_interaction_ < 0.001; Figure 2]. Among individuals who never smoked or drank alcohol regularly, the frequency of spicy food intake was significantly inversely associated with the risks of oesophageal (*p*_trend_ < 0.0001) and colorectal cancer (*p*_trend_ = 0.006) (Figure 3). The inverse associations of spicy food intake with oesophageal cancer were apparent in female never-regular smokers and never-regular drinkers, but there were too few cases among male never-regular smokers and never-regular drinkers for reliable assessment (Supplementary Figures 2 and 3, available as Supplementary data at *IJE* online). For oesophageal cancer, the inverse association with spicy food consumption appeared somewhat stronger in the high-risk region (i.e. Huixian, *p*_trend_ = 0.005) than in the other regions combined, but the number of cases involved in the regular-consumption group was small in the high-risk region (Supplementary Table 6, available as Supplementary data at *IJE* online).

Sensitivity analyses with additional adjustment for adiposity or region stratification were generally consistent with the main results **(**Supplementary Tables 7 and 8, available as Supplementary data at *IJE* online). Further exclusion of participants from areas with extreme intake (Hunan and Sichuan), with prior ulcers, or with any prior chronic diseases and the first 3 years of follow-up did not materially change the inverse association between spicy food intake frequency and oesophageal cancer risk, but associations with stomach and colorectal cancers were attenuated to the null (Supplementary Figures 4 and 5, available as Supplementary data at *IJE* online).

**Figure 4:**
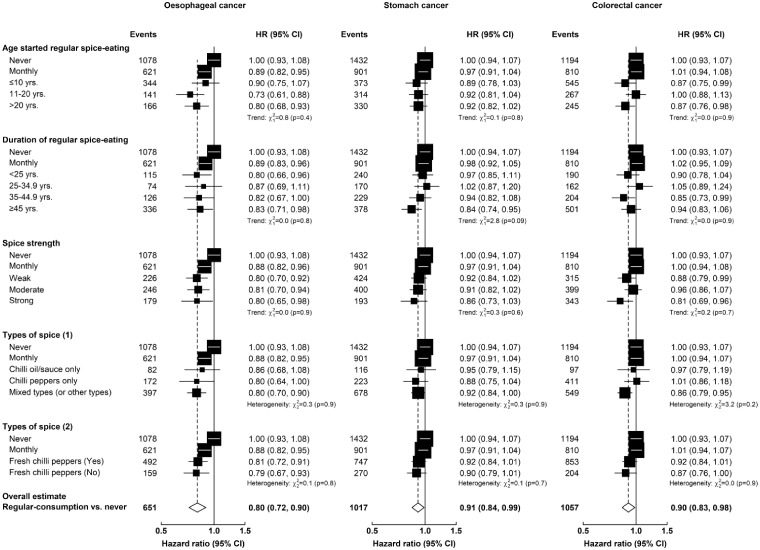
Adjusted hazard ratios (HRs) for gastrointestinal-tract cancers by other spicy food consumption patterns (in regular consumers). Analyses were stratified by age-at-risk (10-year bands) and sex, and adjusted for regions, education level, household-income level, family history of cancer, smoking status, alcohol consumption, physical activity and consumption of fruits, meat, dairy and preserved vegetables (plus tea consumption and temperature for oesophageal cancer). Analyses for duration were additionally adjusted for baseline age. Tests for trend or heterogeneity were conducted within regular consumers only. (This plot was not adjusted for frequency within regular consumers, but additional adjustment for frequency made no material change.)

Amongst regular consumers of spicy food, the risks of the three cancers did not differ significantly by starting age or duration of regular spicy food intake; by strength of spicy food usually preferred/used; or by types of spicy food normally consumed (Figure 4).

## Discussion

To our knowledge, our study is the first study to prospectively assess the associations of spicy food consumption with the risks of incident GI cancers. In this Chinese adult population, about one-third reported daily/almost daily consumption of spicy food. Overall, a higher frequency of spicy food consumption was significantly associated with a lower risk of GI cancers, especially oesophageal cancer, and this inverse association was restricted mainly to never-regular smokers, never-regular drinkers and those who never regularly smoked or drank. Similar, though somewhat weaker, inverse associations were also found for stomach and colorectal cancers.

Previous reports on associations of spicy food consumption with GI cancers were all small case–control studies and, in contrast to our findings, they tended to show null or positive associations. A recent meta-analysis of six case–control studies (five from Asia) that included 2009 oesophageal cancer cases found a non-significant increased risk (pooled-OR = 1.43, 95% CI 0.92–2.22) in individuals in the ‘highest’ spicy food intake category compared with those in the ‘lowest’ category.^6^ However, this pooled estimate may be misleading due to recall bias in case–control studies and the incompatibility of the spicy food assessment methods across the studies meta-analysed (with some only assessing frequency^14^^,^^19^^,^^29^ and others only intensity^21^^,^^30^), many of which were crude and/or subjective (e.g. ‘seldom’ vs ‘often’).

To date, only two case–control studies on oesophageal cancer—one in Australia (844 cases) and another in India (236 cases)—had detailed quantitative assessment of spicy food intake.^15^^,^^29^ Whereas the Australian study found no association with the frequency of spicy food consumption,^29^ the Indian study reported a significant positive dose–response relationship with the amount of red chilli powder intake.^15^ Neither examined this association specifically among never-regular smokers and/or never-regular drinkers to minimize residual confounding. The present prospective study included more oesophageal cancer cases (*N* = 2350) than those included in the meta-analysis and found a highly significant inverse association with spicy food consumption frequency, which appeared to be restricted mainly to those who did not smoke and/or drink alcohol regularly. As smoking and alcohol-drinking are both strong risk factors for oesophageal cancer (particularly ESCC),^31–33^ any protective effects associated with spicy food may be masked by the large excess risk associated with these factors, especially since frequent consumers of spicy food in CKB were more likely to be smokers and/or drinkers. This may help to partially explain the absence of any apparent protective association of spicy food consumption in ever-regular smokers and/or alcohol-drinkers in the present study. In this study, over half of the oesophageal cancer cases occurred in one high-risk area, namely Huixian,^34^ for reasons that are still unclear. Despite this, associations observed between spicy food consumption and oesophageal cancer risk were directionally consistent in high-risk and other regions.

For stomach cancer, previous case–control studies reported either null or positive associations with spicy food intake,^6^ of which only three studies (conducted in Mexico and Korea) had quantitative assessment of consumption, comprehensive adjustment for confounders and >200 cases of stomach cancer.^17^^,^^18^^,^^35^ With almost 15 times the number of stomach cancer cases, we found a weak inverse association that was attenuated towards the null after excluding the first 3 years of follow-up, suggesting that the inverse association may be due in part or wholly to reverse causation. As for colorectal cancer, only three case–control studies were identified, of which two (each with <200 cases) reported positive associations.^36^^,^^37^ The largest study, with 400 cases and conducted in Sichuan, however, found no significant association.^38^ Similarly to stomach cancer, the weak inverse association with colorectal cancer in our study appeared to be partly explained by reverse causation, to be confirmed in other large prospective studies. Since no previous study has examined spicy food intake with sub-sites of stomach and colorectal cancers, further evidence is needed to determine whether associations truly differ between cardia and non-cardia stomach cancer, and between colon and rectal cancer.

Capsaicin, the main bioactive constituent of spicy food, has exhibited various carcinogenic effects in animal studies, e.g. through inducing mucosal damage.^39^ In contrast, capsaicin has also demonstrated anti-carcinogenic effects, through altering GI cancer risk factors such as inhibiting the growth of *Helicobacter pylori* (*H. pylori*)^40^ and reducing body fat.^41^^,^^42^ Specifically for adiposity, cross-sectional epidemiological studies in China have also reported inverse associations of chilli intake with prevalence of obesity and serum cholesterol levels^12^^,^^13^ but these were not replicated in CKB and we also did not find clear evidence of mediation by adiposity. Overall, it is possible that any carcinogenicity or anti-carcinogenicity of capsaicin is dependent on dose, and there may be a threshold beyond which the harms start to outweigh the benefits (or vice versa), but further epidemiological studies with quantitative assessment are needed to clarify this.

The strengths of our study included the prospective design, large numbers of cases, assessment of multiple aspects of spicy food intake and adjustment for a wide range of confounders. However, limitations exist. First, spicy food consumption was self-reported and we did not have objective indicators such as capsaicin concentration extracted from participants’ food to validate self-reported preferences for spice strength. To mitigate this issue and to better distinguish between mild-, moderate- and strong-spice consumers, interviewers were instructed to monitor the coherence of participants’ answers across the different spicy food questions (since daily spice consumers or those who consume chillies directly tend to prefer stronger spice) and to clarify with participants when there were important contradictions. Second, the quantity of spicy food intake was not available for more accurate quantification of the observed relationships or investigation of potential threshold effects. Third, we were unable to explore the effects of spice type and strength in the two regions where consumption levels were very high (Hunan and Sichuan) as participants in these regions consumed almost exclusively fresh chilli, at moderate/high intensity. Although the high consumption levels in these two regions might have distorted the overall associations, our sensitivity analyses restricted to the other eight areas showed broadly consistent results with the main findings. Fourth, although we have attempted to control for a wide range of known and suspected confounders, residual confounding from age, regions (e.g. urban–rural differences), suboptimally measured factors (e.g. other dietary factors) or unmeasured factors (e.g. *H. pylori* infection for stomach cancer) may still be present. For example, residual confounding from age and urban–rural residency, if any, could have biased the associations with oesophageal and stomach cancers away from and towards the null, respectively. Nonetheless, there were no significant subgroup differences by urban–rural areas (*p*_heterogeneity_ > 0.05) and associations remained directionally-consistent after excluding individuals with a prior history of peptic ulcers (a proxy for symptomatic *H. pylori* infection).^43^ Finally, even as the single largest study on this topic, we had limited power to draw conclusions on the interaction between spicy food intake and smoking or alcohol-drinking.

In conclusion, among Chinese adults, the frequency of spicy food consumption was associated with a lower risk of total GI cancers, particularly oesophageal cancer, and this inverse relationship was much more pronounced among those who did not smoke and drink alcohol regularly and did not appear to differ by types of spicy food used. Relationships between spicy food consumption and risks of stomach and colorectal cancers were less clear.

## Supplementary data

Supplementary data are available at *IJE* online.

## Author contributions

W.C.C. conducted the literature review and data analyses, and drafted the manuscript under the supervision of L.Y., I.M. and Z.C. W.C.C., L.Y., I.M. and Z.C. revised the manuscript. C.K., H.D. and R.W. provided methodological support. Y.G., Z.B., P.H. and C.H. were involved in data collection/management. Y.C., J.L., L.L. and Z.C. were involved in funding and data acquisition. All authors had opportunities to review and approve the manuscript.

## Funding

This work was supported by the Kadoorie Charitable Foundation in Hong Kong for the baseline and first resurvey; and the UK Wellcome Trust (088158/Z/09/Z, 104085/Z/14/Z), the Chinese Ministry of Science and Technology (2011BAI09B01, 2012–14), the Chinese National Natural Science Foundation (81390540, 81390541, 81390544) and the National Key Research and Development Program of China (grants 2016YFC0900500, 2016YFC0900501, 2016YFC0900504, 2016YFC1303904) for the long-term continuation of the CKB cohort. The British Heart Foundation, Medical Research Council and Cancer Research UK provided the core funding to the Clinical Trial Service Unit and Epidemiological Studies Unit of the University of Oxford.

## Supplementary Material

dyaa275_Supplementary_Data
