# Supplementary material for: Spicy food consumption and risk of gastrointestinal-tract cancers: findings from the China Kadoorie Biobank
Source: Int J Epidemiol. 2021 Jan 23;50(1):199–211. doi: 10.1093/ije/dyaa275 (PMC7938514; doi:10.1093/ije/dyaa275)
Supplement: dyaa275_Supplementary_Data [file dyaa275_supplementary_data.pdf]

Table S1. Reproducibility of spicy food questions

	Weighted Kappa values
	Baseline and re-assessment within 12 months (N = 923)
Spicy food frequency	0.54
Spicy food starting age	0.60
Spicy food strength	0.49
Spicy food types	0.37

Table S2. Mean (95%CI) levels of adiposity and blood biochemical markers by frequency of spicy food intake *

		Frequency of spicy food consumption									
		**Never/ rarely**		**Monthly**		**1-2 days/wk**		**3-5 days/wk**		**6-7 days/wk**	
	**N**	**N**	**Mean (95%CI)**	**N**	**Mean (95%CI)**	**N**	**Mean (95%CI)**	**N**	**Mean (95%CI)**	**N**	**Mean (95%CI)**
**Adiposity measures**											
Body Mass Index, kg/m^2	510099	166972	23.33 (23.30 - 23.36)	126923	23.51 (23.48 - 23.55)	32934	23.63 (23.58 - 23.67)	29677	23.76 (23.71 - 23.80)	153593	23.87 (23.84 - 23.91)
Body Fat, %	510101	166931	26.16 (26.10 - 26.22)	126856	26.51 (26.44 - 26.57)	32901	26.71 (26.62 - 26.80)	29658	26.90 (26.81 - 26.99)	153516	27.19 (27.12 - 27.26)
Waist Circumference, cm	510101	166972	80.88 (80.79 - 80.97)	126923	81.37 (81.28 - 81.46)	32934	81.72 (81.59 - 81.84)	29677	81.91 (81.78 - 82.04)	153595	82.24 (82.15 - 82.34)
Waist-to-Hip Ratio	510101	166972	0.89 (0.89 - 0.89)	126923	0.89 (0.89 - 0.89)	32934	0.89 (0.89 - 0.89)	29677	0.89 (0.89 - 0.89)	153595	0.90 (0.89 - 0.90)
**Blood biochemistry (in subset) †**											
HDL-C (mmol/L)	6338	1734	1.24 (1.21 - 1.26)	1258	1.24 (1.22 - 1.26)	334	1.25 (1.22 - 1.29)	355	1.23 (1.19 - 1.27)	2657	1.25 (1.22 - 1.28)
LDL-C (mmol/L)	6338	1734	2.42 (2.36 - 2.47)	1258	2.42 (2.37 - 2.47)	334	2.38 (2.31 - 2.46)	355	2.40 (2.32 - 2.48)	2657	2.42 (2.37 - 2.48)
Triglyceride (mmol/L) ‡	6338	1734	1.68 (1.60 - 1.76)	1258	1.72 (1.64 - 1.81)	334	1.79 (1.67 - 1.93)	355	1.71 (1.58 - 1.84)	2657	1.76 (1.67 - 1.86)
Total Cholesterol (mmol/L)	6338	1734	4.75 (4.68 - 4.82)	1258	4.76 (4.69 - 4.84)	334	4.77 (4.66 - 4.88)	355	4.71 (4.60 - 4.83)	2657	4.78 (4.70 - 4.86)
ApoA1 (mg/dL)	6327	1730	135.50 (133.81 - 137.19)	1254	135.06 (133.31 - 136.80)	333	136.86 (134.29 - 139.42)	355	134.63 (132.04 - 137.22)	2655	136.37 (134.51 - 138.24)
ApoB (mg/dL)	6338	1734	86.04 (84.47 - 87.62)	1258	86.49 (84.86 - 88.12)	334	85.20 (82.81 - 87.59)	355	85.27 (82.86 - 87.68)	2657	85.91 (84.17 - 87.64)
Lp(a) (mmol/L) ‡	6079	1670	23.0 (21.0 - 25.1)	1203	22.9 (20.9 - 25.1)	322	22.2 (19.5 - 25.4)	342	23.2 (20.3 - 26.6)	2542	23.3 (21.1 - 25.7)
CRP (mg/L) ‡	6325	1730	0.98 (0.89 - 1.08)	1256	1.06 (0.96 - 1.17)	333	1.07 (0.93 - 1.24)	355	1.08 (0.93 - 1.24)	2651	1.00 (0.90 - 1.11)
Fibrinogen (g/L)	3517	932	3.17 (3.10 - 3.25)	699	3.10 (3.02 - 3.18)	192	3.14 (3.03 - 3.26)	216	3.21 (3.09 - 3.32)	1478	3.13 (3.05 - 3.21)
Albumin (g/L)	5808	1579	42.38 (42.15 - 42.60)	1156	42.39 (42.16 - 42.62)	300	42.61 (42.27 - 42.94)	333	42.48 (42.14 - 42.82)	2440	42.41 (42.17 - 42.65)

Abbreviations: CI, confidence intervals; HDL-C, high-density lipoprotein cholesterol; LDL-C, low-density lipoprotein cholesterol; ApoA1, Apolipoprotein A1; ApoB, Apolipoprotein B; CRP, C-reactive protein.
* Analyses were adjusted for age, sex, study area, education levels, household income, smoking, alcohol consumption, physical activity, and intake of fruits, meat, dairy products and preserved vegetables.
† Blood biomarker data are only available in a subset of participants and those with missing or invalid measures were excluded.
‡ Geometric means (95%CIs) are presented.

Table S3. Other measures of spicy food consumption, by consumption frequency in regular-consumers*

	Frequency of spicy food consumption		
**Other spicy food measures**	**1-2 days/week (N=32,933)**	**3-5 days/week (N=29,677)**	**6-7 days/week (N=153,594)**
Age started regular spicy food intake, %			
≤10 y	21.7	25.0	34.7
11-20.9 y	31.2	30.2	27.6
21-39.9 y	30.4	28.1	22.3
≥40 y	16.8	16.7	15.4
Duration of regular spicy food intake, %			
<25 y	47.1	44.5	37.5
25-34.9 y	20.2	19.7	21.6
35-44.9 y	15.1	18.0	19.6
≥45 y	17.6	17.9	21.2
Spice strength, %			
Mild	70.4	56.4	28.9
Moderate	26.2	32.7	40.9
Strong	3.3	10.9	30.2
Main source of spicy food, %			
Only chilli oil or sauce	14.8	10.3	6.9
Only chilli peppers (either fresh or dried)	32.5	31.1	29.5
Mixed types or other types	52.8	58.7	63.6

*Data on other spicy food measures were only collected in regular spicy food consumers (i.e. those who eat at least once per week). Percentages were directly standardised to the age, sex and region structure of the overall CKB population.

Table S4. Hazard ratios (HRs) for incident GI cancer subtypes or subsites, according to spicy food consumption frequency*

	Frequency of spicy food consumption								
	Never/ rarely (N=166,972)		Monthly (N=126,923)		1-5 days/wk (N=32,934)		6-7 days/wk (N=153,595)		
	Events	HR (95%CI)	Events	HR (95%CI)	Events	HR (95%CI)	Events	HR (95%CI)	P-trend
**Oesophageal Squamous †**	351	1.00 (0.88 -1.14)	194	0.86 (0.75 -0.99)	58	0.92 (0.70 -1.21)	123	0.81 (0.62 -1.04)	0.1
**Stomach Cardia**	280	1.00 (0.87 -1.15)	154	0.93 (0.80 -1.08)	36	0.68 (0.49 -0.95)	49	0.77 (0.55 -1.07)	0.04
**Stomach Non-cardia**	1152	1.00 (0.93 -1.07)	747	0.99 (0.92 -1.06)	359	0.97 (0.88 -1.08)	573	0.91 (0.82 -1.02)	0.2
**Colon**	601	1.00 (0.91 -1.10)	423	1.08 (0.99 -1.19)	171	1.01 (0.86 -1.17)	354	1.10 (0.95 -1.28)	0.4
**Rectum**	618	1.00 (0.91 -1.10)	405	0.95 (0.86 -1.04)	160	0.83 (0.71 -0.97)	387	0.74 (0.63 -0.88)	0.0009

* Analyses were stratified by age-at-risk (10-year bands) and sex, and adjusted for regions, education level, household income level, family history of cancer, smoking status, alcohol consumption, and physical activity (MET-hours/day), dietary factors (consumption of fruits, meat, dairy, preserved vegetables) (plus tea consumption and temperature for oesophageal cancer)

† Data from the cancer-adjudication dataset. All oesophageal cancer cases that have not been confirmed to be oesophageal squamous cell cancer were excluded from this analysis.

Table S5. Adjusted HRs for GI cancer mortality according to spicy food consumption frequency*

	Frequency of spicy food consumption					
	Never/ rarely (N=166,972)	Monthly (N=126,923)	1-2 days/wk (N=32,934)	3-5 days/wk (N=29,677)	6-7 days/wk (N=153,595)	P-trend
**Oesophageal Cancer**						
No. of deaths	574	339	50	53	306	
Hazard ratio (95% CI)	1.00 (0.90 -1.11)	0.91 (0.82 -1.01)	0.80 (0.61 -1.06)	0.93 (0.71 -1.23)	0.79 (0.67 -0.94)	0.02
**Stomach Cancer**						
No. of deaths	720	478	116	108	371	
Hazard ratio (95% CI)	1.00 (0.91 -1.10)	1.01 (0.93 -1.10)	1.00 (0.83 -1.20)	1.02 (0.84 -1.24)	0.91 (0.80 -1.04)	0.3
**Colorectal Cancer**						
No. of deaths	338	264	57	57	263	
Hazard ratio (95% CI)	1.00 (0.88 -1.14)	1.17 (1.04 -1.32)	1.08 (0.83 -1.40)	1.22 (0.94 -1.59)	1.00 (0.83 -1.20)	0.9

* Analyses were stratified by age-at-risk (10-year bands) and sex, and adjusted for study area, education level, household income level, family history of cancer, smoking status, alcohol consumption, physical activity (MET-hours/day), and dietary factors (consumption of fruits, meat, dairy, preserved vegetables) (plus tea consumption and temperature for oesophageal cancer)

Table S6. Associations of spicy food intake with oesophageal cancer (EC) risk, stratified by high-risk and other regions*

	High-risk (Huixian)			Other regions		
	EC cases	HR (95%CI)	P-trend‡	EC cases	HR (95%CI)	P-trend‡
**Spicy food**						
Never or rarely	727	1.00 (0.92 -1.08)		351	1.00 (0.88 -1.14)	
Monthly	385	0.90 (0.82 -1.00)		236	0.94 (0.83 -1.06)	
1-5 days/week	32	0.72 (0.51 -1.02)		133	0.88 (0.74 -1.04)	
6-7 days/week	44	0.71 (0.53 -0.96)	0.005	442	0.89 (0.77 -1.03)	0.2

Abbreviation: EC, oesophageal cancer;
* Categories have been collapsed to maintain statistical power. Analyses were stratified by age-at-risk (10-year bands) and sex, and adjusted for study area, education level, household income level, family history of cancer, smoking status, alcohol consumption, physical activity (MET-hours/day), dietary factors (consumption of fruits, meat, dairy, preserved vegetables), and tea consumption and temperature

Table S7. Hazard ratios for GI cancers by spicy food consumption frequency, with additional adjustment for adiposity *

	Frequency of spicy food consumption					
	Never/ rarely (N=166,972)	Monthly (N=126,923)	1-2 days/wk (N=32,934)	3-5 days/wk (N=29,677)	6-7 days/wk (N=153,595)	P-trend
**Oesophageal Cancer**						
No. of events	1078	621	83	82	486	
Hazard ratio (95% CI)†	1.00 (0.93 -1.08)	0.89 (0.83 -0.96)	0.77 (0.62 -0.95)	0.85 (0.68 -1.06)	0.82 (0.72 -0.94)	0.004
Hazard ratio (95% CI)‡	1.00 (0.93 -1.08)	0.89 (0.82 -0.96)	0.76 (0.61 -0.95)	0.84 (0.68 -1.05)	0.82 (0.72 -0.94)	0.003
Hazard ratio (95% CI)§	1.00 (0.93 -1.08)	0.89 (0.83 -0.96)	0.77 (0.62 -0.95)	0.85 (0.68 -1.06)	0.83 (0.72 -0.94)	0.004
**Stomach Cancer**						
No. of events	1432	901	212	183	622	
Hazard ratio (95% CI)†	1.00 (0.94 -1.07)	0.98 (0.92 -1.04)	0.96 (0.84 -1.10)	0.93 (0.80 -1.08)	0.91 (0.82 -1.00)	0.09
Hazard ratio (95% CI)‡	1.00 (0.94 -1.07)	0.98 (0.92 -1.04)	0.96 (0.83 -1.10)	0.93 (0.80 -1.08)	0.90 (0.82 -1.00)	0.08
Hazard ratio (95% CI)§	1.00 (0.94 -1.07)	0.98 (0.92 -1.04)	0.96 (0.84 -1.10)	0.93 (0.80 -1.08)	0.91 (0.82 -1.01)	0.1
**Colorectal Cancer**						
No. of events	1194	810	180	144	733	
Hazard ratio (95% CI)†	1.00 (0.93 -1.07)	1.00 (0.93 -1.07)	0.94 (0.81 -1.09)	0.85 (0.72 -1.01)	0.89 (0.79 -0.99)	0.02
Hazard ratio (95% CI)‡	1.00 (0.93 -1.07)	1.00 (0.93 -1.07)	0.93 (0.81 -1.08)	0.85 (0.72 -1.00)	0.88 (0.79 -0.98)	0.01
Hazard ratio (95% CI)§	1.00 (0.93 -1.07)	1.00 (0.94 -1.07)	0.94 (0.81 -1.09)	0.85 (0.72 -1.01)	0.89 (0.80 -1.00)	0.03

*Abbreviation: wk, week;
† Adjusted for covariates in Model 3 (see Table 2) plus body mass index
‡ Adjusted for covariates in Model 3 plus waist circumference
§ Adjusted for covariates in Model 3 plus body fat percentage

Table S8. Hazard ratios for GI cancers by spicy food consumption frequency, with region-stratification*

	Frequency of spicy food consumption				
	Never/ rarely (N=166,972)	Monthly (N=126,923)	1-5 days/wk (N=62,611)	6-7 days/wk (N=153,595)	P-trend
**Oesophageal Cancer**					
No. of events	1078	621	165	486	
Hazard ratio (95% CI)†	1.00 (0.93 -1.08)	0.91 (0.84 -0.98)	0.82 (0.70 -0.96)	0.81 (0.71 -0.93)	0.002
**Stomach Cancer**					
No. of events	1432	901	395	622	
Hazard ratio (95% CI)†	1.00 (0.94 -1.07)	0.97 (0.91 -1.03)	0.93 (0.84 -1.02)	0.89 (0.80 -0.99)	0.05
**Colorectal Cancer**					
No. of events	1194	810	324	733	
Hazard ratio (95% CI)†	1.00 (0.93 -1.07)	1.01 (0.94 -1.08)	0.92 (0.82 -1.03)	0.91 (0.81 -1.02)	0.09

*Abbreviation: wk, week;

Analyses were stratified by age-at-risk (10-year bands), sex and regions, and adjusted for education level, household income level, family history of cancer, smoking status, alcohol consumption, physical activity (MET-hours/day), and dietary factors (consumption of fruits, meat, dairy, preserved vegetables) (plus tea consumption and temperature for oesophageal cancer)

Figure S1. Frequency of spicy food consumption by CKB study areas*

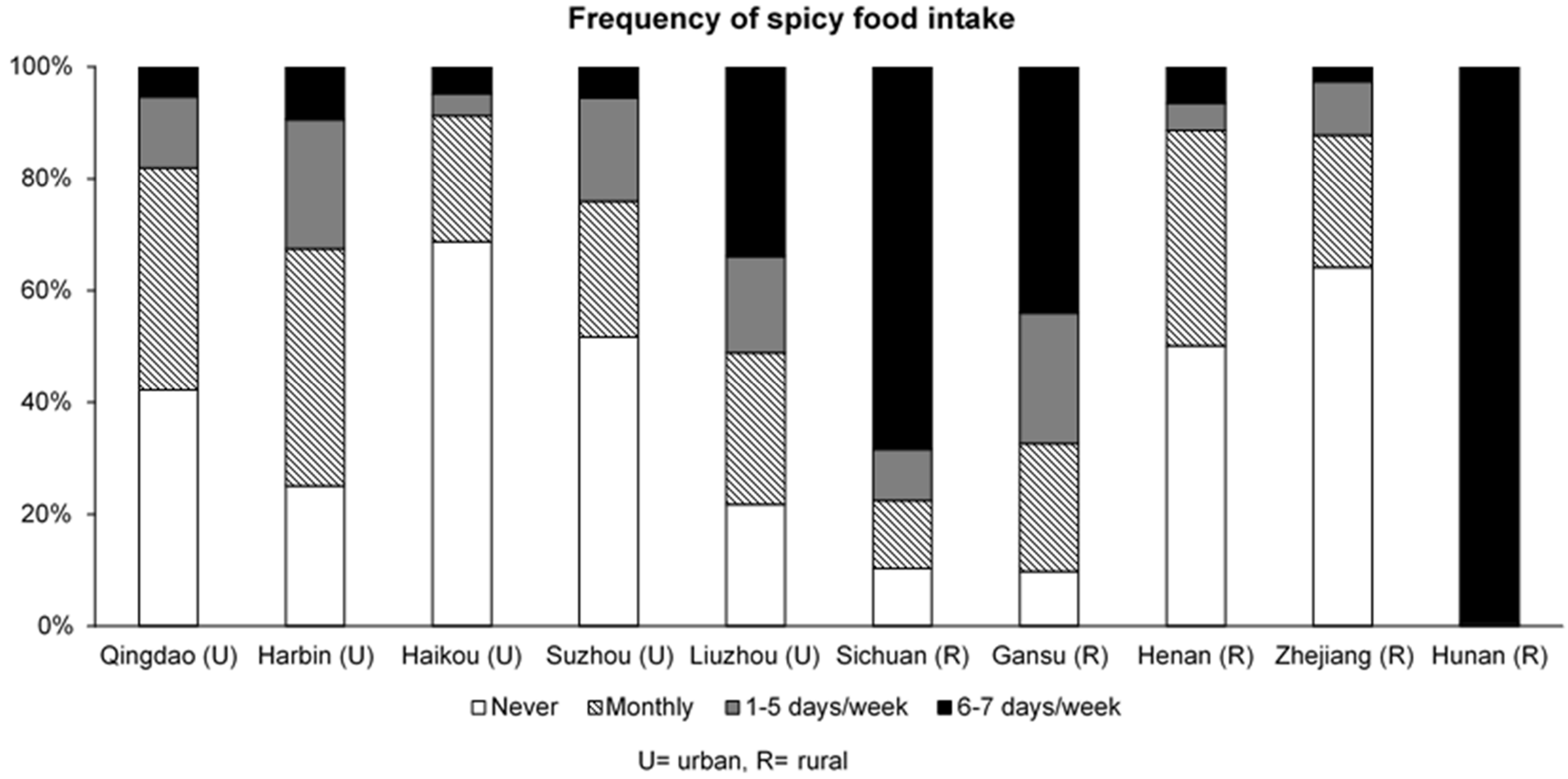

* standardised by age and sex

Figure S2. Adjusted Hazard Ratios (HRs) for GI cancers by frequency of spicy food consumption in FEMALE never-regular smokers and MALE never-regular and ever-regular smokers

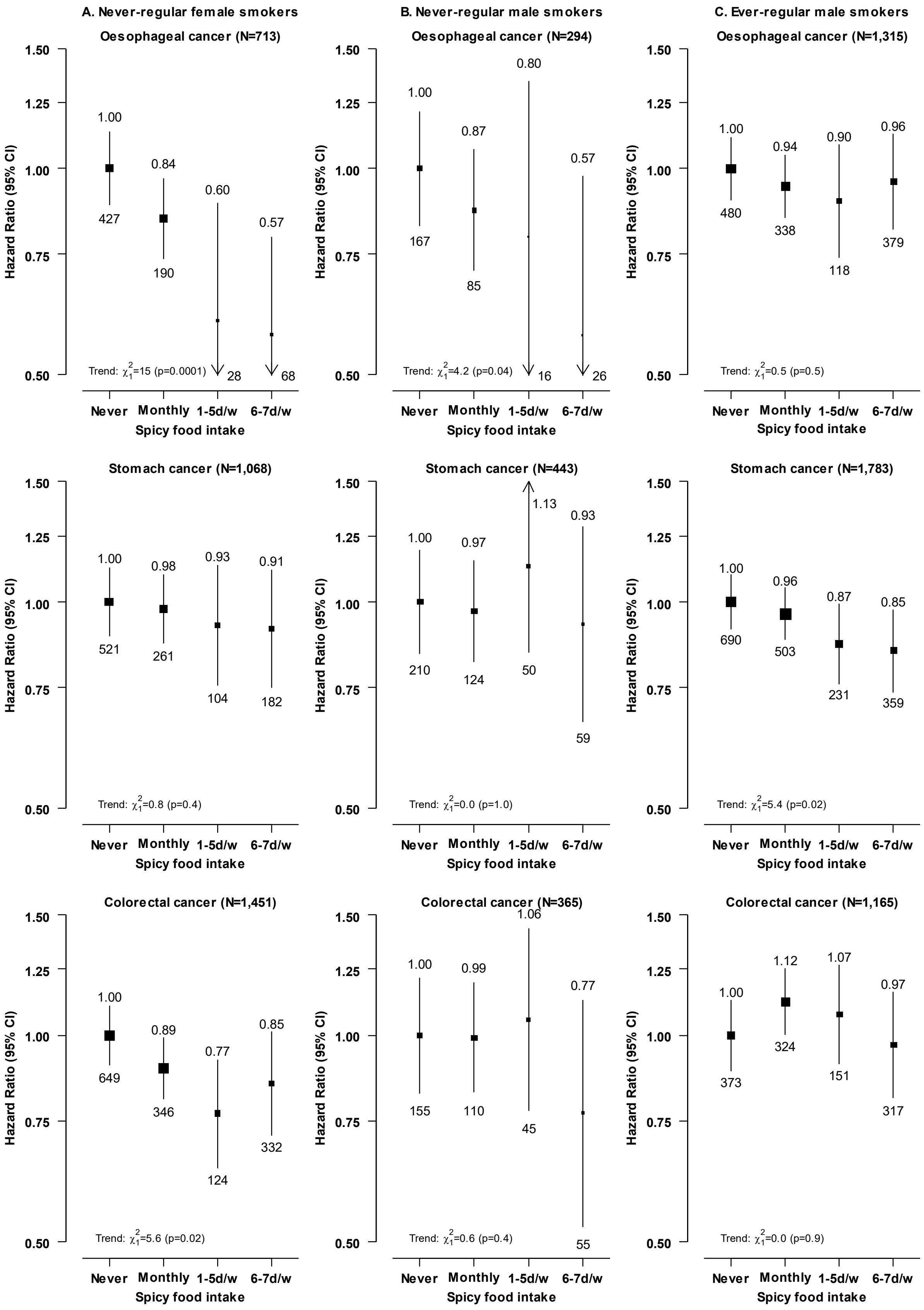

Analyses were stratified by age-at-risk (10-year bands), adjusted for regions, education level, household income level, family history of cancer, alcohol consumption, physical activity, consumption of fruits, meat, dairy, preserved vegetables (plus tea consumption and temperature for oesophageal cancer).

Figure S3. Adjusted Hazard Ratios (HRs) for GI cancers by frequency of spicy food consumption in FEMALE never-regular drinkers and MALE never-regular and ever-regular drinkers

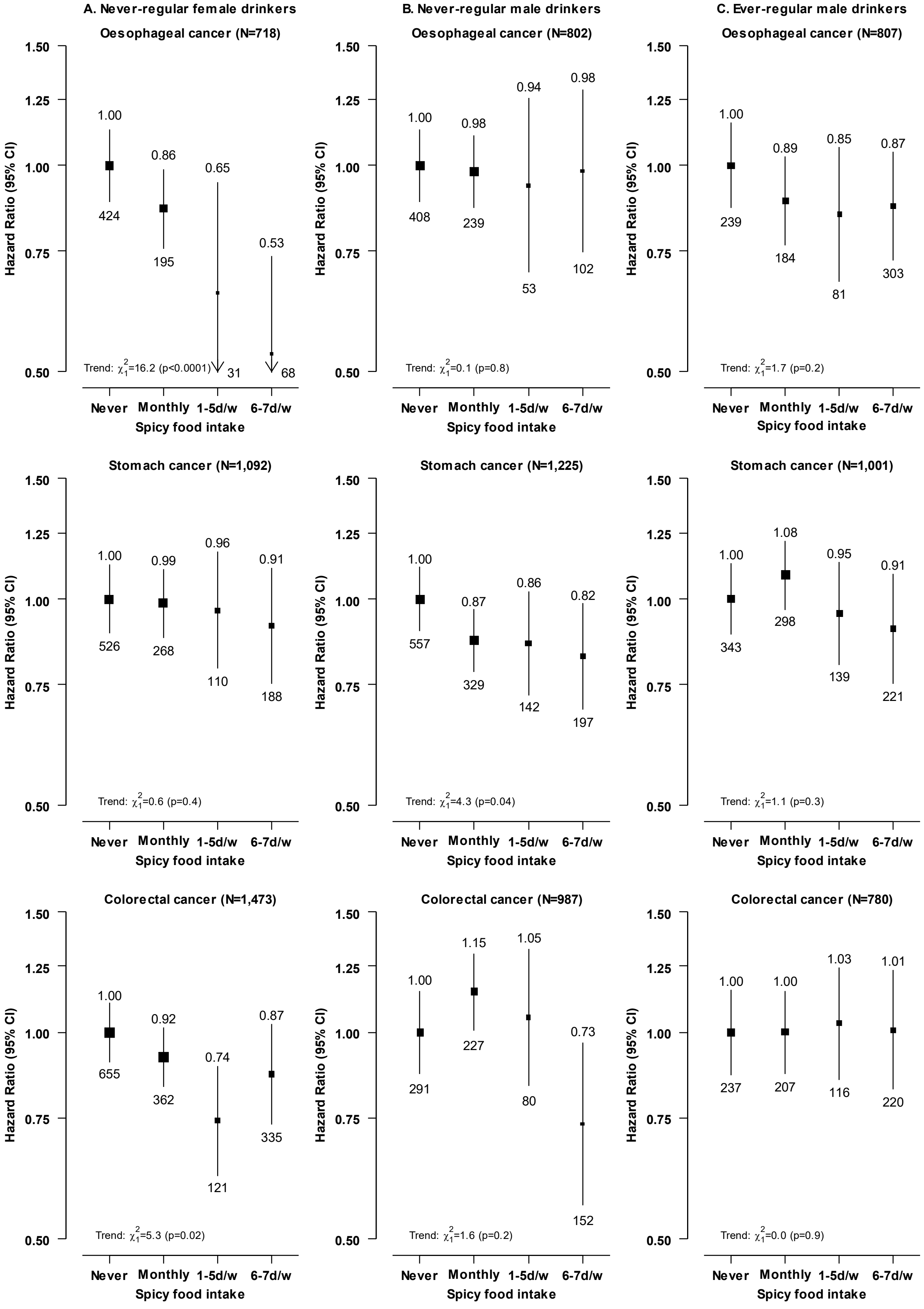

Analyses were stratified by age-at-risk (10-year bands), adjusted for regions, education level, household income level, family history of cancer, smoking status, physical activity, consumption of fruits, meat, dairy, preserved vegetables (plus tea consumption and temperature for oesophageal cancer).

Figure S4. Adjusted Hazard Ratios (HRs) for GI cancers by frequency of spicy food consumption, after excluding Hunan and Sichuan

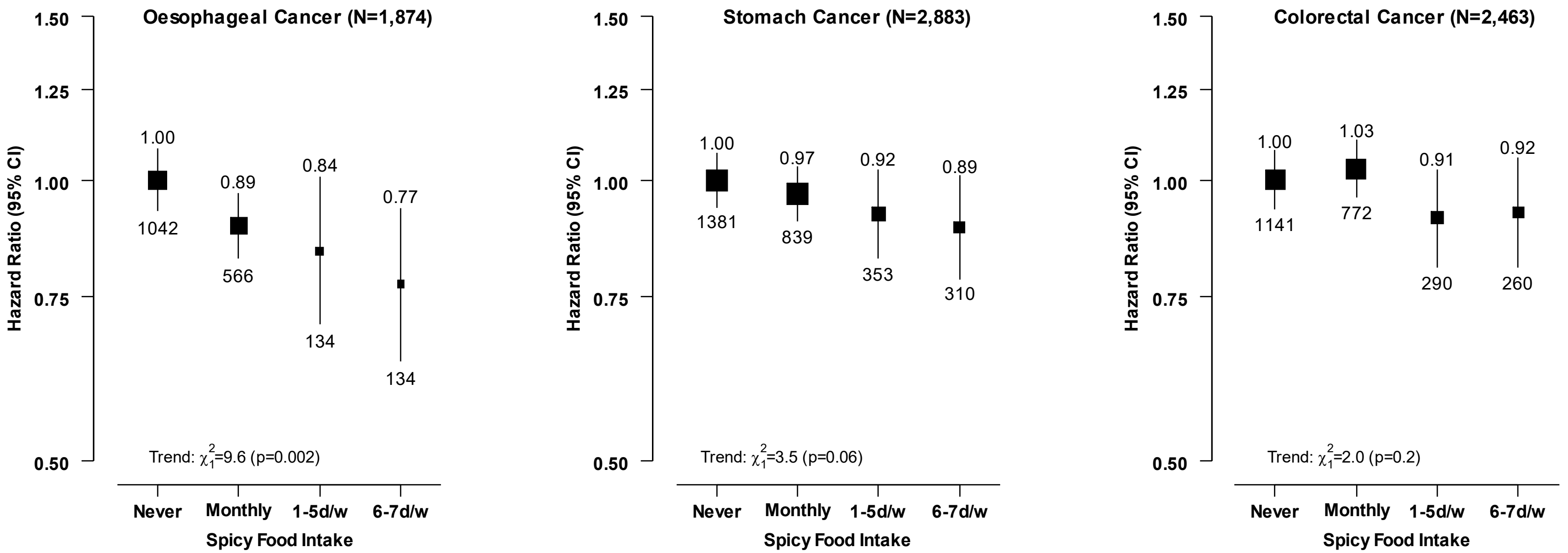

Analyses were stratified by age-at-risk (10-year bands) and sex, and adjusted for regions, education level, household income level, family history of cancer, smoking status, alcohol consumption, physical activity, consumption of fruits, meat, dairy, preserved vegetables (plus tea consumption and temperature for oesophageal cancer).

Figure S5. Adjusted Hazard Ratios (HRs) for GI cancers by spicy food consumption frequency, excluding (a) prior peptic ulcers and (b) any prior diseases* and first 3 years of follow-up

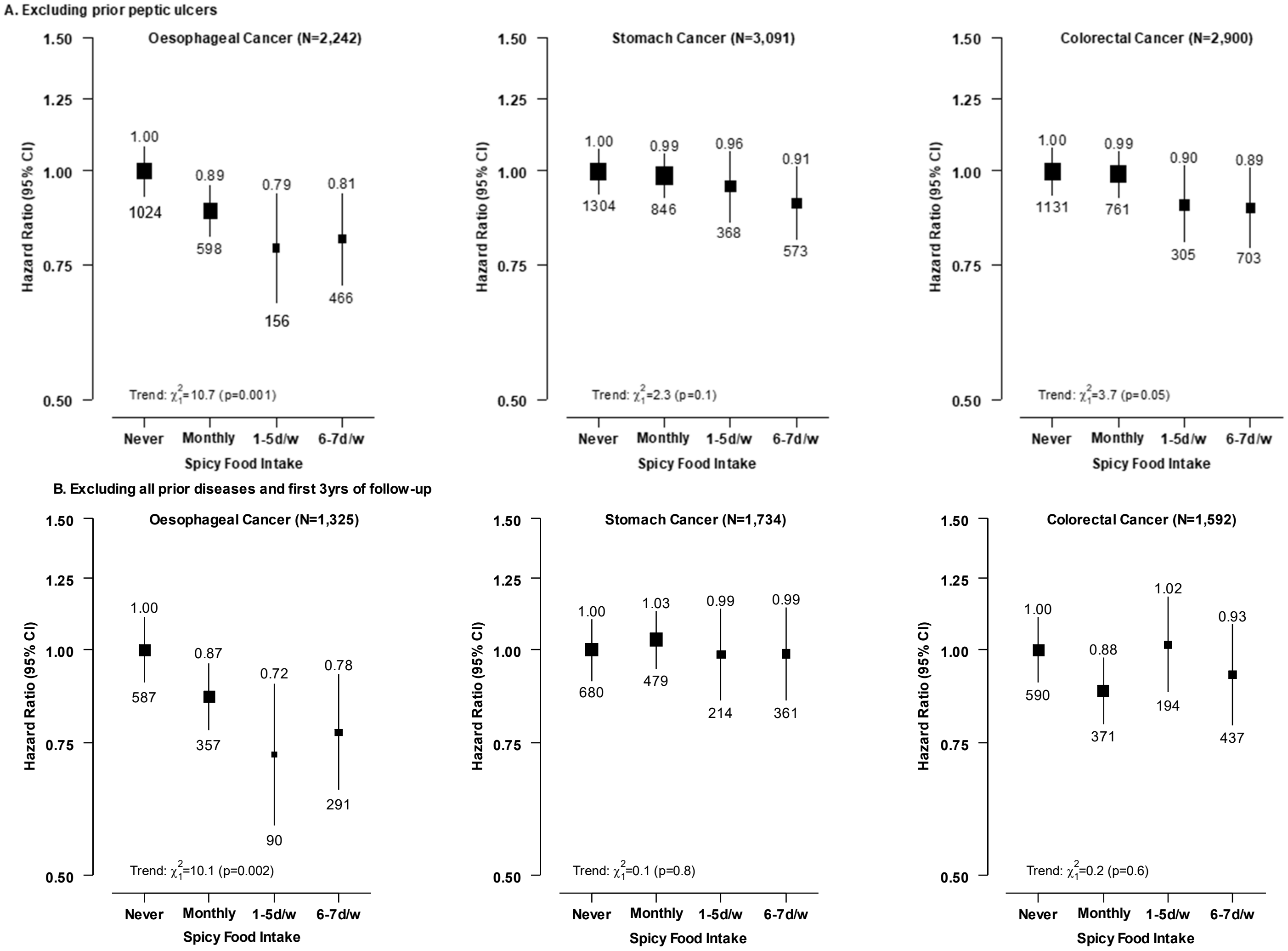

Prior diseases included chronic heart diseases, stroke/transient ischemic attack, hypertension, diabetes, peptic ulcers, cirrhosis and kidney diseases (participants with prior cancer were not included in this study). Analyses were stratified by age-at-risk (10-year bands) and sex, and adjusted for regions, education level, household income level, family history of cancer, smoking status, alcohol consumption, physical activity, consumption of fruits, meat, dairy, preserved vegetables (plus tea consumption and temperature for oesophageal cancer).
